# Alcohol and drug use during unprotected anal intercourse among gay and bisexual men in Scotland: what are the implications for HIV prevention?

**DOI:** 10.1136/sextrans-2013-051195

**Published:** 2013-12-17

**Authors:** Jessica Li, Lisa M McDaid

**Affiliations:** 1 Section of Public Health, University of Sheffield, Sheffield, UK; 2 MRC/CSO Social and Public Health Sciences Unit, University of Glasgow, Glasgow, UK

**Keywords:** Gay Men, Sexual Behaviour, Drug Misuse, HIV

## Abstract

**Objectives:**

To examine alcohol and drug use during unprotected anal intercourse (UAI), and whether use is associated with HIV-related risk behaviours among gay and bisexual men in Scotland.

**Methods:**

Cross-sectional survey of 17 gay commercial venues in Glasgow and Edinburgh in May 2011 (n=1515, 65.2% response rate); 639 men reporting UAI are included.

**Results:**

14.4% were always and 63.4% were sometimes drunk during UAI in the previous 12 months; 36.3% always/sometimes used poppers; 22.2% always/sometimes used stimulant or other recreational/illicit drugs; and 14.1% always/sometimes used Viagra. All were significantly correlated and, in multivariate analysis, the adjusted odds of having UAI with 2+ partners in the previous 12 months were significantly higher for men who reported stimulant or recreational/illicit drug use during UAI (AOR=2.75, 95% CI 1.74 to 4.34) and the adjusted odds of UAI with casual partners were higher for men who reported poppers use (AOR=1.50, 95% CI 1.03 to 2.17). Men who reported always being drunk during UAI were more likely to report UAI with 2+ partners (AOR=1.68, 95% CI 1.01 to 2.81), casual partners (AOR=2.18, 95% CI 1.27 to 3.73), and partners of unknown/discordant HIV status (AOR=2.14, 95% CI 1.29 to 3.53), than men who were not.

**Conclusions:**

Our study suggests alcohol and drug use may be relatively common during UAI among gay and bisexual men in Scotland. Brief alcohol or drug interventions, particularly in clinical settings, are justified, but should be properly evaluated and take into account the potential influence of broader, situational and social factors on sexual risk.

## Introduction

With continuing and increasing HIV incidence among men who have sex with men (MSM) in the UK,^1^ ongoing research is required to determine which factors are associated with HIV-related sexual risk behaviours and could be amenable to intervention. The Scottish and English Sexual Health Frameworks identify MSM and those who have alcohol and drug problems as higher-risk groups for poor sexual health outcomes,^2^ ^3^ and there is growing focus on the rise in alcohol consumption and related health problems in the UK. In response to these issues, The Royal College of Physicians and British Association for Sexual Health and HIV has recommended that sexual health settings should distribute information on alcohol-related harms and could facilitate brief alcohol interventions to reduce consumption and related sexual ill health.^4^

Some studies have found alcohol and substance use to be significant factors in HIV acquisition among MSM,^5^ with reported links between alcohol and drug use and HIV-related sexual risk behaviour (such as unprotected anal intercourse (UAI)^5^ ^6^), HIV infection^7^ ^8^ and sexually transmitted infections (STI).^9^ Although a number of studies have focused primarily on intravenous drug use,^10^ ^11^ an emphasis on non-injecting drug use should also be considered because research suggests gay and bisexual populations are more likely to report higher use of alcohol and drugs than the general population.^12^ ^13^ Despite over two decades of research, the association between substance use and HIV remains contested, and while current research is dominated by American studies, evidence within Britain is somewhat limited.^5^ ^14^ In the 2007 UK Gay Men's Sex Survey, 85% of men reported using alcohol in the last year, 42% had used poppers, 21% had used Ecstasy, and 21% had used cocaine,^15^ and the National LGB Drug & Alcohol Database identified alcohol, poppers and cannabis as the most commonly used drugs among MSM.^16^ We found only one study specific to Scotland, conducted in 1998, which reported that men who used cannabis or poppers less than two hours before sex were more likely to have unprotected sex.^17^ Recent estimates of alcohol and drug use during sex among MSM in the UK are particularly limited, but a recent Sigma Blast report reported that one-third of participants were always, almost always, or more often than not, using poppers during sex.^13^ In this paper, we examine the frequency of alcohol and drug use during UAI among gay and bisexual men in Scotland, and whether alcohol and drug use during UAI is associated with particular HIV-related sexual risk behaviours.

## Methods

The 2011 MRC Gay Men's Sexual Health Survey collected anonymous, self-complete questionnaires and (Orasure) oral fluid specimens in 17 gay commercial venues (15 bars and 2 saunas) in Glasgow and Edinburgh in May 2011. This represents the majority of exclusively gay venues in each city. A form of time and location sampling was used to recruit representative samples of gay and bisexual men using the venues. Temporary fieldworkers distributed questionnaires at two different time points in the early (19.00–21.00) and late evening (21.00–23.00). No bar was visited twice in the same evening and at the end of the survey, each bar had been visited at both time points on each day of the week. Each sauna was visited six times over the course of the 2-week survey period; for a 2 h period between 17:00–19:00 on Thursdays and between 14:00–17:00 on Saturdays and Sundays. All men present or entering the venues were asked to participate; 1515 of the men approached participated in the survey (65.2% response rate (RR)) and 1218 provided oral fluid samples (52.4% RR). Oral fluid specimens were analysed at the West of Scotland Specialist Virology Centre (screened for anti-HIV using an enzyme immunoassay; positives rescreened, and repeat reactives confirmed using western blot). Ethical approval was granted by University of Glasgow College of Social Sciences ethics committee.

Questionnaires included demographics (age, area of residence, employment status, sexual orientation, education and frequency of gay scene use), sexual health (HIV and STI testing) and sexual behaviour (number of sexual, anal and UAI partners, partner type and HIV status) in the previous 12 months. Men were asked “Thinking about the times you had anal sex WITHOUT a condom in the last 12 months, how often were you (a) drunk on alcohol; (b) using poppers; (c) using Viagra or other erectile dysfunction drugs; (d) using stimulant drugs (eg, speed, cocaine, etc); (e) using other recreational or illicit drugs (eg, ecstasy, ketamine)”, with response options of always, sometimes and never. Responses were recoded into binary drug use categories (always/sometimes vs never) for multivariate analyses due to the small number of cases in some of the always categories.

Men who reported having no UAI in the previous 12 months (n=810) and men who reported having UAI in the previous 12 months but had missing data on any of the alcohol or drug use and UAI variables (n=66) were excluded from the analyses, giving a sample of 639 men. After adjusting for survey location, sexual orientation, age, survey venue, postcode, employment status, qualifications, gay scene use, HIV/STI testing, HIV treatment optimism and HIV status, when compared with excluded men in multivariate analysis, the included sample had higher odds of identifying as gay (AOR=2.44, 95% CI 1.60 to 3.72) compared with bisexual, being aged 16–25 years (AOR=2.35, 95% CI 1.63 to 3.37) and 26–35 (AOR=1.89, 95% CI 1.32 to 2.70) compared with 45+ years, visiting the gay scene at least once a week (AOR=1.29, 95% CI 1.00 to 1.67) compared with once a month or less, having an HIV or STI test in the previous 12 months (AOR=1.34, 95% CI 1.07 to 1.67), and providing an oral fluid sample and testing HIV negative (AOR=1.82, 95% CI 1.38 to 2.42).

χ^2^ tests were used for bivariate comparisons. Multivariate logistic regression models were used to estimate OR and 95% CIs for HIV-related sexual risk behaviour (UAI with two or more partners, UAI with casual partners, and UAI with partners of unknown or discordant HIV status). We adjusted for factors significant at the bivariate level (p<0.05), (a) alcohol, (b) poppers, (c) Viagra or other erectile dysfunction drugs and (d) stimulant or recreational/illicit drugs, and any factors associated with these. Stimulant and recreational/illicit drug use categories were combined because of significant correlations found between them (r_s_(637)=0.777, p<0.001). All alcohol and drug use variables were included in the logistic regression models because of significant associations between them. Regression models were constructed using the default method (Forced Entry Method) in SPSS 15.0 for Windows.

## Results

### Sample characteristics

The average age of participants was 31 years (range 18–68, SD=10.07) and the majority of men identified as gay (94.7%); 42.6% were surveyed in Edinburgh and 57.4% were surveyed in Glasgow; most (76.6%) resided in either city or its surrounding area. Degree or postgraduate education was reported by 45.6%, further/vocational education by 38.6%, and secondary only by 15.9%. Most men were frequent visitors of the gay scene (48.5% attended at least once a week and 24.6% attended 2–3 times a month). The majority disagreed with, or were uncertain about, the two HIV treatment optimism statements: ‘I am less worried about HIV infection now that treatments have improved’; ‘I believe that new drug therapies make people with HIV less infectious’ (79.3% and 86.6%, respectively). Almost two-thirds (63.1%) reported having had either an HIV or STI test in the previous 12 months; 24 men tested HIV positive (3.8%) and the remainder of those who provided an oral fluid sample tested HIV negative; 91 did not provide an oral fluid sample, of whom 91.2% self-reported as HIV negative. In terms of HIV-related sexual risk behaviours, 28.0% reported 2+ UAI partners, 52.7% reported UAI with casual partners, and 55.1% reported UAI with partners of unknown/discordant status in the previous 12 months.

### Alcohol and drug use

Overall, there was a high level of alcohol and drug use during UAI in the previous 12 months among this sample. Alcohol was the most frequently used drug; 497 men (77.8%) reported ever being drunk during UAI (14.4% reported they were always drunk, 63.4% were sometimes drunk). Always or sometimes using poppers was reported by 232 men (36.3%); 22 (3.4%) always used and 210 (32.9%) sometimes used these. Overall, 142 men (22.2%) reported always or sometimes using stimulant, recreational or other illegal drugs. Within this category, 13 men (2.0%) reported that they always used stimulant drugs and 98 (15.3%) sometimes used these; 11 men (1.7%) reported always using recreational or other illicit drugs, and 117 (18.3%) reported sometimes using these. Viagra was the least frequently reported; only 3 men (0.5%) reported always using it and 87 (13.6%) said that they sometimes used it.

There were significant associations between the alcohol and drug use variables; 39.8% of men who used alcohol during UAI also reported using poppers compared with 23.9% of men who never used alcohol (χ^2^=12.07, p=0.001); 27.0% of men using alcohol had used stimulant or recreational/illicit drugs compared with 5.6% of men who never used it (χ^2^=29.07, p<0.001). Significant associations were also found among Viagra and other drug use. Over half of those who used Viagra reported using poppers compared with one-third of those who never used Viagra (χ^2^=27.87, p<0.001), and half of those using Viagra also used stimulant or recreational/illicit drugs compared with one-fifth of those who never used it (χ^2^=50.58, p<0.001). Finally, those using stimulant or recreational/illicit drugs were more likely to use alcohol (χ^2^=29.07, p<0.001), poppers (χ^2^=43.8, p<0.001), and Viagra (χ^2^=50.58, p<0.001).

### Factors associated with alcohol and drug use

Factors associated with alcohol and drug use during UAI in the previous 12 months are shown in table 1. Younger men, those who resided in Glasgow or Edinburgh, and those who frequently visited the gay scene were more likely to report always or sometimes using alcohol. Older men and men who were HIV positive, were more likely to report always or sometimes using poppers. HIV positive men were also more likely to report always or sometimes using a stimulant or other recreational/illicit drug during UAI. Men who were older, more optimistic about HIV treatments and HIV positive, were more likely to have always or sometimes used Viagra. The adjusted odds of all these factors except for both HIV treatment optimism measures remained significant in multivariate analyses for all alcohol/drug use categories (data not shown).

**Table 1:** Factors associated with alcohol and drug use during unprotected anal intercourse in the previous 12 months: n, row % (n=639)

	Drunk on alcohol			Used poppers			Used stimulant or recreational/illicit drugs			Used Viagra		
	Never, n (%)	Always/sometimes, n (%)	p Value	Never, n (%)	Always/sometimes, n (%)	p Value	Never, n (%)	Always/sometimes, n (%)	p Value	Never, n (%)	Always/sometimes, n (%)	p Value
Overall	142 (22.2)	497 (77.8)		407 (63.7)	232 (36.3)		497 (77.8)	142 (22.2)		549 (85.9)	90 (14.1)	
Survey location												
Edinburgh	66 (24.3)	206 (75.7)	0.285	168 (61.8)	104 (38.2)	0.383	216 (79.4)	56 (20.6)	0.392	234 (86.0)	38 (14.0)	0.943
Glasgow	76 (20.7)	291 (79.3)		239 (65.1)	128 (34.9)		281 (76.6)	86 (23.4)		315 (85.8)	52 (14.2)	
Sexual orientation												
Gay	133 (22.1)	469 (77.9)	0.844	382 (63.5)	220 (36.5)	0.621	472 (78.4)	130 (21.6)	0.284	520 (86.4)	82 (13.6)	0.255
Bisexual	8 (23.5)	26 (76.5)		23 (67.6)	11 (32.4)		24 (70.6)	10 (29.4)		27 (79.4)	7 (20.6)	
Age (years)												
16–25	43 (18.5)	189 (81.5)	<0.001	163 (70.3)	69 (29.7)	0.035	180 (77.6)	52 (22.4)	0.167	222 (95.7)	10 (4.3)	<0.001
26–35	38 (17.3)	182 (82.7)		138 (62.7)	82 (37.3)		172 (78.2)	48 (21.8)		196 (89.1)	24 (10.9)	
36–45	36 (29.0)	88 (71.0)		70 (56.5)	54 (43.5)		90 (72.6)	34 (27.4)		88 (71.0)	36 (29.0)	
45+	25 (40.3)	37 (59.7)		35 (56.5)	27 (43.5)		54 (87.1)	8 (12.9)		42 (67.7)	20 (32.3)	
Area of residence												
Elsewhere	46 (31.7)	99 (68.3)	0.001	91 (62.8)	54 (37.2)	0.763	121 (83.4)	24 (16.6)	0.072	126 (86.9)	19 (13.1)	0.617
Glasgow/Edinburgh	90 (19.0)	384 (81.0)		304 (64.1)	170 (35.9)		362 (76.4)	112 (23.6)		404 (85.2)	70 (14.8)	
Employment												
Employed	114 (22.2)	399 (77.8)	1.00	319 (62.2)	194 (37.8)	0.109	404 (78.8)	109 (21.2)	0.232	437 (85.2)	76 (14.8)	0.284
Other	28 (22.2)	98 (77.8)		88 (69.8)	38 (30.2)		93 (73.8)	33 (26.2)		112 (88.9)	14 (11.1)	
Education												
Secondary	20 (21.1)	75 (78.9)	0.860	65 (68.4)	30 (31.6)	0.561	72 (75.8)	23 (24.2)	0.837	81 (85.3)	14 (14.7)	0.262
Further/vocational	48 (20.8)	183 (79.2)		147 (63.6)	84 (36.4)		182 (78.8)	49 (21.2)		206 (89.2)	25 (10.8)	
Degree/postgraduate	62 (22.7)	211 (77.3)		170 (62.3)	103 (37.7)		212 (77.7)	61 (22.3)		230 (84.2)	43 (15.8)	
Frequency of gay scene use												
Once a month or less	56 (32.6)	116 (67.4)	<0.001	117 (68.0)	55 (32.0)	0.299	143 (83.1)	29 (16.9)	0.071	140 (81.4)	32 (18.6)	0.084
2–3 times a month	34 (21.7)	123 (78.3)		94 (59.9)	63 (40.1)		124 (79.0)	33 (21.0)		141 (89.8)	16 (10.2)	
Once or more a week	52 (16.8)	258 (83.2)		196 (63.2)	114 (36.8)		230 (74.2)	80 (25.8)		268 (86.5)	42 (13.5)	
HIV treatment optimism 1*												
Agree	32 (25.0)	96 (75.0)	0.409	80 (62.5)	48 (37.5)	0.827	95 (74.2)	33 (25.8)	0.287	103 (80.5)	25 (19.5)	0.045
Uncertain/disagree	106 (21.6)	385 (78.4)		312 (63.5)	179 (36.5)		386 (78.6)	105 (21.4)		429 (87.4)	62 (12.6)	
HIV treatment optimism 2*												
Agree	21 (25.3)	62 (74.7)	0.479	53 (63.9)	30 (36.1)	0.915	58 (69.9)	25 (30.1)	0.066	65 (78.3)	18 (21.7)	0.032
Uncertain/disagree	117 (21.8)	419 (78.2)		339 (63.2)	197 (36.8)		423 (78.9)	113 (21.1)		467 (87.1)	69 (12.9)	
Had either an HIV or STI test in previous 12 months												
No	56 (24.1)	176 (75.9)	0.354	150 (64.7)	82 (35.3)	0.749	189 (81.5)	43 (18.5)	0.083	204 (87.9)	28 (12.1)	0.322
Yes	83 (21.0)	313 (79.0)		251 (63.4)	145 (36.6)		299 (75.5)	97 (24.5)		337 (85.1)	59 (14.9)	
HIV status (from saliva test)												
HIV−	116 (22.1)	408 (77.9)	0.945	342 (65.3)	182 (34.7)	0.049	420 (80.2)	104 (19.8)	<0.001	461 (88.0)	63 (12.0)	<0.001
HIV+	6 (25.0)	18 (75.0)		10 (41.7)	14 (58.3)		11 (45.8)	13 (54.2)		12 (50.0)	12 (50.0)	
Did not provide oral fluid specimen	20 (22.0)	71 (78.0)		55 (60.4)	36 (39.6)		66 (72.5)	25 (27.5)		76 (83.5)	15 (16.5)	

*HIV treatment optimism 1—‘I am less worried about HIV infection now that treatments have improved’, HIV treatment optimism 2—‘I believe that new drug therapies make people with HIV less infectious’.

### Factors associated with HIV-related sexual risk behaviour

Tables 2–4 show factors associated with UAI with 2+ partners, casual partners and partners of unknown/discordant status in the previous 12 months.

**Table 2:** Factors associated with unprotected anal intercourse with two or more partners in the previous 12 months: n, row %, unadjusted and multivariate logistic regression (n=639)

	Yes, n (%)	OR	95% CI	p Value	AOR*	95% CI	p Value
Overall	179 (28.0)						
Survey location							
Edinburgh	66 (24.3)	1					
Glasgow	113 (30.8)	1.39	0.97 to 1.98	0.070			
Sexual orientation							
Gay	164 (27.2)	1			1		
Bisexual	15 (44.1)	2.11	1.05 to 4.25	0.037	1.81	0.85 to 3.86	0.124
Age†							
16–25	65 (28.0)	1		0.392	1		0.582
26–35	57 (25.9)	0.90	0.59 to 1.36		0.96	0.61 to 1.52	
36–45	34 (27.4)	0.97	0.60 to 1.58		0.90	0.51 to 1.59	
45+	23 (37.1)	1.52	0.84 to 2.73		1.46	0.74 to 2.89	
Area of residence‡							
Elsewhere	32 (22.1)	1			1		
Glasgow/Edinburgh	143 (30.2)	1.53	0.98 to 2.37	0.059	1.18	0.72 to 1.91	0.514
Employment							
Employed	137 (26.7)	1					
Other	42 (33.3)	1.37	0.90 to 2.09	0.139			
Education							
Secondary	25 (26.3)	1.06	0.62 to 1.80				
Further/vocational	73 (31.6)	1.37	0.93 to 2.02				
Degree/postgraduate	69 (25.3)	1		0.441			
Frequency of gay scene use							
Once a month or less	29 (16.9)	1		<0.001	1		0.002
2–3 times a month	42 (26.8)	1.80	1.06 to 3.07		2.00	1.11 to 3.61	
Once or more a week	108 (34.8)	2.64	1.66 to 4.19		2.60	1.54 to 4.39	
HIV treatment optimism 1§							
Agree	44 (34.4)	1					
Uncertain/disagree	129 (26.3)	0.68	0.45 to 1.03	0.070			
HIV treatment optimism 2§							
Agree	28 (33.7)	1					
Uncertain/disagree	145 (27.1)	0.73	0.45 to 1.19	0.208			
Had either an HIV or STI test in previous 12 months							
No	43 (18.5)	1			1		
Yes	130 (32.8)	2.15	1.45 to 3.18	<0.001	1.89	1.24 to 2.86	0.003
HIV status (from saliva test)							
HIV−	150 (28.6)	1		0.010	1		0.003
HIV+	12 (50.0)	2.49	1.10 to 5.67		1.52	0.59 to 3.91	
Did not provide oral fluid specimen	17 (18.7)	0.57	0.33 to 1.00		0.48	0.26 to 0.88	
Drunk on alcohol¶							
Never	35 (24.6)	1			1		
Always/sometimes	144 (29.0)	1.25	0.81 to 1.91	0.312	0.81	0.49 to 1.33	0.397
Used poppers							
Never	95 (23.3)	1			1		
Always/sometimes	84 (36.2)	1.86	1.31 to 2.65	0.001	1.44	0.96 to 2.14	0.076
Used stimulant or recreational/illicit drugs							
Never	111 (22.3)	1			1		
Always/sometimes	68 (47.9)	3.20	2.16 to 4.73	<0.001	2.75	1.74 to 4.34	<0.001
Used Viagra							
Never	138 (25.1)	1			1		
Always/sometimes	41 (45.6)	2.49	1.58 to 3.94	<0.001	1.70	0.97 to 2.98	0.062

*Adjusted for sexual orientation, age, area of residence, frequency of gay scene use, HIV/STI testing, HIV status, alcohol, popper, stimulant or recreational/illicit drug and Viagra use.

†Age—Associated with always or sometimes using alcohol, poppers or Viagra during UAI in the previous 12 months (see table 1).

‡Area of residence—Associated with always or sometimes using alcohol during UAI in the previous 12 months (see table 1).

§HIV treatment optimism 1—‘I am less worried about HIV infection now that treatments have improved’, HIV treatment optimism 2—‘I believe that new drug therapies make people with HIV less infectious’.

¶Drunk on alcohol—All alcohol and drug use variables were included in the logistic regression models because of significant correlations found between them.

**Table 3:** Factors associated with unprotected anal intercourse with casual partners in the previous 12 months: n, row %, unadjusted and multivariate logistic regression (n=639)

	Yes, n (%)	OR	95% CI	p Value	AOR*	95% CI	p Value
Overall	337 (52.7)						
Survey location							
Edinburgh	126 (46.3)	1			1		
Glasgow	211 (57.5)	1.57	1.14 to 2.15	0.005	1.48	1.05 to 2.11	0.028
Sexual orientation							
Gay	311 (51.7)	1			1		
Bisexual	25 (73.5)	2.60	1.19 to 5.66	0.016	2.17	0.94 to 5.02	0.071
Age							
16 to 25	146 (62.9)	1		0.001	1		0.036
26–35	105 (47.7)	0.54	0.37 to 0.78		0.62	0.41 to 0.94	
36–45	57 (46.0)	0.50	0.32 to 0.78		0.58	0.35 to 0.96	
45+	28 (45.2)	0.49	0.28 to 0.86		0.47	0.24 to 0.89	
Area of residence†							
Elsewhere	69 (47.6)	1			1		
Glasgow/Edinburgh	256 (54.0)	1.29	0.89 to 1.88	0.176	1.09	0.72 to 1.66	0.681
Employment							
Employed	266 (51.9)	1					
Other	71 (56.3)	1.20	0.81 to 1.78	0.365			
Education							
Secondary	52 (54.7)	1.61	1.01 to 2.58		1.42	0.85 to 2.36	
Further/vocational	142 (61.5)	2.13	1.49 to 3.04		1.90	1.29 to 2.81	
Degree/postgraduate	117 (42.9)	1		<0.001	1		0.009
Frequency of gay scene use							
Once a month or less	71 (41.3)	1		<0.001	1		0.001
2–3 times a month	74 (47.1)	1.27	0.82 to 1.96		1.20	0.74 to 1.93	
Once or more a week	192 (61.9)	2.32	1.58 to 3.39		2.12	1.38 to 3.26	
HIV treatment optimism 1‡							
Agree	103 (44.4)	1			1		
Uncertain/disagree	227 (57.3)	0.64	0.43 to 0.96	0.029	0.81	0.51 to 1.30	0.382
HIV treatment optimism 2‡							
Agree	78 (60.9)	1			1		
Uncertain/disagree	246 (50.1)	0.61	0.38 to 0.99	0.045	0.52	0.30 to 0.92	0.026
Had either an HIV or STI test in previous 12 months							
No	52 (62.7)	1			1		
Yes	272 (50.7)	1.68	1.21 to 2.33	0.002	1.56	1.09 to 2.23	0.016
HIV status (from saliva test)							
HIV–	286 (54.6)	1		0.044	1		0.168
HIV+	14 (58.3)	1.17	0.51 to 2.67		0.84	0.34 to 2.09	
Did not provide oral fluid specimen	37 (40.7)	0.57	0.36 to 0.90		0.62	0.38 to 1.02	
Drunk on alcohol							
Never	63 (44.4)	1			1		
Always/sometimes	274 (55.1)	1.54	1.06 to 2.24	0.024	1.19	0.78 to 1.83	0.423
Used poppers							
Never	201 (49.4)	1			1		
Always/sometimes	136 (58.6)	1.45	1.05 to 2.01	0.025	1.50	1.03 to 2.17	0.034
Used stimulant or recreational/illicit drugs							
Never	248 (49.9)	1			1		
Always/sometimes	89 (62.7)	1.69	1.15 to 2.47	0.007	1.21	0.77 to 1.90	0.421
Used Viagra§							
Never	283 (51.5)	1			1		
Always/sometimes	54 (60.0)	1.41	0.90 to 2.22	0.138	1.50	0.86 to 2.60	0.150

*Adjusted for survey location, sexual orientation, age, area of residence, employment, education, frequency of gay scene use, HIV treatment optimism 1+2, HIV/STI testing, HIV status, alcohol, popper, stimulant or recreational/illicit drug and Viagra use.

†Area of residence—Associated with always or sometimes using alcohol during UAI in the previous 12 months (see table 1).

‡HIV treatment optimism 1—‘I am less worried about HIV infection now that treatments have improved’, HIV treatment optimism 2—‘I believe that new drug therapies make people with HIV less infectious’.

§Used Viagra—All alcohol and drug use variables were included in the logistic regression models because of significant correlations found between them.

**Table 4:** Factors associated with unprotected anal intercourse with unknown or discordant HIV status partners in the previous 12 months: n, row %, unadjusted and multivariate logistic regression (n=639)

	Yes, n (%)	OR	95% CI	p Value	AOR*	95% CI	p Value
Overall	352 (55.1)						
Survey location							
Edinburgh	131 (48.2)	1			1		
Glasgow	221 (60.2)	1.63	1.19 to 2.24	0.003	1.67	1.20 to 2.33	0.003
Sexual orientation							
Gay	330 (54.8)	1					
Bisexual	21 (61.8)	1.33	0.66 to 2.71	0.429			
Age							
16–25	121 (52.2)	1		0.029	1		0.101
26–35	111 (50.5)	0.93	0.65 to 1.35		0.89	0.61 to 1.32	
36–45	77 (62.1)	1.50	0.96 to 2.35		1.38	0.85 to 2.24	
45+	42 (67.7)	1.93	1.07 to 3.48		1.78	0.94 to 3.37	
Area of residence†							
Elsewhere	81 (55.9)	1			1		
Glasgow/Edinburgh	260 (54.9)	0.96	0.66 to 1.40	0.831	0.93	0.62 to 1.38	0.707
Employment							
Employed	277 (54.0)	1					
Other	75 (59.5)	1.25	0.84 to 1.86	0.264			
Education							
Secondary	51 (53.7)	1.04	0.65 to 1.66				
Further/vocational	129 (55.8)	1.13	0.80 to 1.61				
Degree/postgraduate	144 (52.7)	1		0.240			
Frequency of gay scene use†							
Once a month or less	88 (51.2)	1		0.254	1		0.151
2–3 times a month	83 (52.9)	1.07	0.69 to 1.65		1.13	0.72 to 1.79	
Once or more a week	181 (58.4)	1.34	0.92 to 1.95		1.47	0.98 to 2.21	
HIV treatment optimism 1‡							
Agree	72 (56.3)	1					
Uncertain/disagree	269 (54.8)	0.94	0.64 to 1.40	0.767			
HIV treatment optimism 2‡							
Agree	47 (56.6)	1					
Uncertain/disagree	294 (54.9)	0.93	0.58 to 1.48	0.762			
Had either an HIV or STI test in previous 12 months							
No	148 (63.8)	1			1		
Yes	196 (49.5)	0.56	0.40 to 0.78	<0.001	0.52	0.36 to 0.73	<0.001
HIV status (from saliva test)							
HIV−	285 (54.4)	1		0.069			
HIV+	19 (79.2)	3.19	1.17 to 8.66				
Did not provide oral fluid specimen	48 (52.7)	0.94	0.60 to 1.46				
Drunk on alcohol§							
Never	75 (52.8)	1			1		
Always/sometimes	277 (55.7)	1.13	0.77 to 1.64	0.538	1.10	0.73 to 1.66	0.652
Used poppers							
Never	211 (51.8)	1			1		
Always/sometimes	141 (60.8)	1.44	1.04 to 2.00	0.029	1.27	0.89 to 1.82	0.184
Used stimulant or recreational/illicit drugs§							
Never	264 (53.1)	1			1		
Always/sometimes	88 (62.0)	1.44	0.98 to 2.11	0.062	1.23	0.79 to 1.89	0.359
Used Viagra							
Never	290 (52.8)	1			1		
Always/sometimes	62 (68.9)	1.98	1.23 to 3.19	0.005	1.58	0.92 to 2.72	0.097

*Adjusted for survey location, age, area of residence, frequency of gay scene use, HIV/STI testing, alcohol, popper, stimulant or recreational/illicit drug and Viagra use.

†Area of residence and frequency of gay scene use – associated with always or sometimes using alcohol during UAI in the previous 12 months (see table 1).

‡HIV treatment optimism 1—‘I am less worried about HIV infection now that treatments have improved’, HIV treatment optimism 2—‘I believe that new drug therapies make people with HIV less infectious’.

§Drunk on alcohol and used stimulant or recreational/illicit drugs—All alcohol and drug use variables were included in the logistic regression models because of significant correlations found between them.

At the bivariate level, the likelihood of having UAI with 2+ partners was significantly higher for men who reported always or sometimes using poppers, stimulant or other recreational/illicit drugs, or Viagra (table 2). In multivariate analysis, the odds of reporting UAI with 2+ partners remained significantly higher for men who reported always or sometimes using a stimulant or recreational/illicit drugs, visited the gay scene at least 2–3 times a month compared with once a month or less, and had a recent HIV or STI test. The odds were lower for men who did not provide an oral fluid specimen (table 2).

Men who reported always or sometimes using alcohol, poppers, or stimulant or recreational/illicit drugs during UAI were more likely to report UAI with casual partners than men who did not (table 3). In multivariate analysis, poppers use remained significant (table 3). The odds of UAI with casual partners were also significantly higher among men who were surveyed in Glasgow, educated to secondary or further/vocational level compared with postgraduate, visited the gay scene at least once a week, and reported having a recent HIV or STI test. The odds were significantly lower among men aged 26+ years and men who were uncertain or did not agree that new drug therapies make people with HIV less infectious.

The likelihood of reporting UAI with partners of unknown/discordant HIV status was higher for men who reported always or sometimes using poppers or Viagra during UAI, but these did not remain significant in multivariate analysis (table 4). Instead, the odds were higher for men surveyed in Glasgow and lower for men who reported a recent HIV or STI test.

Given the relatively higher number of men reporting always being drunk during UAI (14.4%) compared with always using drugs, and current concern around high alcohol consumption, a separate analysis was conducted using the always drunk category compared against sometimes or never. Men who reported always being drunk during UAI were more likely to report UAI with 2+ partners (AOR=1.68, 95% CI 1.01 to 2.81), casual partners (AOR=2.18, 95% CI 1.27 to 3.73), or partners of unknown/discordant status (AOR=2.16, 95% CI 1.31 to 3.56) than men who sometimes or never used alcohol during UAI.

## Discussion

This is the first study to report on alcohol and drug use during UAI among a community-based sample of gay and bisexual men in Scotland since the late 1990s. In 1998, under half the men surveyed in Edinburgh reported alcohol use before or during sex, and less than one in 10 reported using poppers.^17^ In our study, more than three-quarters always or sometimes used alcohol during UAI in the previous 12 months, one-third used poppers, one-fifth used a stimulant or other recreational/illicit drugs, and one in seven used Viagra. This is comparable with general use previously reported in the 2007 UK Gay Men's Sex Survey.^15^ Our findings for popper use are also consistent with those from the recent Sigma Blast report in which one-third of participants reported always, almost always, or more often than not, using poppers during sex.^13^

However, when discussing these results there are some important limitations to our study to consider. Caution should be taken when generalising to the wider population of MSM, given this was a venue-based sample. It has been suggested that samples recruited from bars might contain higher proportions who regularly combine drug use and sex, and engage in risky sexual behaviours in general,^18^ so our estimates could be inflated. Furthermore, the cross-sectional nature of the data precluded any analysis of causality and our estimates could be affected by general recall or reporting bias. The anonymous nature of the survey prevents identification of men who could have completed the questionnaire more than once, but this is thought unlikely given the short timeframe of data collection and the training of fieldworkers to avoid this. General alcohol and drug use among men who did not report UAI was not collected and could not, therefore, be compared with men who reported UAI, and there were significant differences in the characteristics of the men who did and did not report UAI. Men reporting UAI, and included in our analyses, were more likely to identify as gay, were younger, more frequent scene goers, and more likely to have tested for HIV or other STIs in the previous 12 months. It is possible that there could be broader associations between substance use and sexual behaviour more generally, and this is a continuing research gap. Finally, we could not explore men's reasons for drug use. Studies have identified multiple reasons MSM decide to use alcohol or drugs including: psychological and personal (eg, child abuse, gender role confusion, homophobia, social role of alcohol and drugs),^15^ sexual enhancement (eg, methamphetamine and cocaine can heighten sexual desire),^19^ and situational factors (eg, men meeting men in clubs, bars, and other venues where drugs are available).^19^ Furthermore, links between alcohol and drug use and sexual risk behaviours have been widely contested because of methodological limitations which cannot control for confounding factors, such as sensation seeking^20^ ^21^ or the role of social and cultural norms^21^ ^22^; this could not be explored in our study.

Overall, our findings on alcohol and drug use during specific sexual risk events support other recent studies,^11^ ^16^ ^17^ and contribute to the knowledge base in this field. Polydrug use (using two or more drugs in combination) is reportedly common among MSM.^12^ ^23–26^ Here, the associations between drug use categories suggest this could be common among gay and bisexual men in Scotland reporting UAI (although we cannot confirm if drugs were actually used at the same time or at the same UAI risk event). Findings do indicate that there could be a general pattern of widespread alcohol and drug use, whether combined or not, that deserves attention.

Within our study we found that alcohol and drug use differed by age; younger men were more likely to report using alcohol during UAI, while older men were more likely to use poppers or Viagra. This is important to note because there have been fewer studies that have identified risk factors for older MSM compared with younger MSM.^27^ However, one US study reported older MSM were more likely to engage in higher-risk sexual activities after consuming alcohol than younger MSM.^28^ HIV positive men were also more likely to report drug use; a finding that has similarly been reported elsewhere.^12^ ^15^ This is particularly problematic because certain drugs, such as gammahydroxybutrate (GHB) and ketamine, could potentially interfere with antiretroviral medications or adherence.^29^

Research on the association of alcohol and drug use to sexual risk behaviour remains somewhat mixed, and although there has been support for links between sexual risk and methamphetamine and alcohol use, particularly heavy alcohol use, findings for other drugs, such as poppers and Viagra, are more varied.^5^ Our results are similarly mixed. Although there was no significant association between ever using alcohol and the risk behaviours, the association we found between *always* using alcohol during UAI and HIV-related sexual risk behaviours supports past research on links between heavy drinking and increased sexual risk.^5^ ^21^ ^22^ While we found support for associations between stimulant, recreational or other illicit drug use and UAI with 2+ partners, and popper use and UAI with casual partners, we did not find links between drug use and UAI with partners or unknown or discordant status or between Viagra use and any of the risk behaviours. Reasons for the differences remain unclear and of interest. Although HIV-related sexual risk behaviours were associated with one another (eg, more than two-thirds of men engaged in two or more), differential patterns of substance use may reflect differential patterns of specific risk taking within and between the sexual behaviours. This has implications for intervention development and is worthy of further qualitative investigation. Most men reporting HIV-related sexual risk behaviours also reported recent HIV/STI testing; a reassuring association we have noted previously.^30^ This suggests that clinical settings have access to men at high risk of reporting HIV-related behaviours, and as such, are appropriate sites for intervention, which could include a specific focus on alcohol and drug use.

There is limited evidence of the effectiveness of interventions to reduce substance use and associated sexual risk behaviours among MSM.^25^ There is increasing concern about alcohol consumption and related harms to public health, and in the UK, pilot evaluations of brief alcohol interventions are underway in sexual health settings.^3^ ^4^ However, our findings suggest the associations between alcohol and drug use and sexual risk are complex and unlikely to be amenable to straightforward intervention. Additional associations with age, gay scene use, education and location suggest the influence of broader, situational and social factors. Exploring this, and reasons for drug use (as well as polydrug use), in future research would be beneficial and enable a more in-depth understanding of the relationship between alcohol and drug use and HIV-related sexual risk behaviours; an essential step in addressing these issues in future interventions.

**Key messages**

- Alcohol use during UAI was common among gay and bisexual men surveyed in Scotland and substantial minorities reported use of other drugs, particularly poppers.
- Moderate correlations between the drug use categories suggest that polydrug use could be common among men in Scotland reporting UAI.
- Significant associations between alcohol and drug use during UAI and HIV-related risk behaviours were apparent, but inconsistent and mediated by other demographic and behavioural factors.
- Brief alcohol or drug interventions in clinical settings should consider the potential influence of broader, situational and social factors on sexual risk.
